# Neural progenitor cell-derived exosomes in ischemia/reperfusion injury in cardiomyoblasts

**DOI:** 10.1186/s12868-025-00931-1

**Published:** 2025-02-05

**Authors:** Oiva Arvola, Virpi Stigzelius, Minna Ampuja, Riikka Kivelä

**Affiliations:** 1 Division of Intensive Care, Department of Anaesthesiology and Intensive Care, University of Helsinki and Helsinki University Hospital, Helsinki, Finland; 2 Stem Cells and Metabolism Research Program, Research Programs Unit, Faculty of Medicine, University of Helsinki, Helsinki, Finland; 3 Wihuri Research Institute, Helsinki, Finland; 4 Faculty of Sport and Health Sciences, University of Jyväskylä, Jyväskylä, Finland

**Keywords:** Neural stem cells, Exosome, Small EV, Stroke, Myocardial infarction, IR-injury

## Abstract

The physiologic relationship between the brain and heart is emerging as a novel therapeutic target for clinical intervention for acute myocardial infarction. In the adult human brain, vestigial neuronal progenitor stem cells contribute to neuronal repair and recovery following cerebral ischemic injury, an effect modulated by secreted exosomes. Ischemia conditioned neuronal cell derived supernatant and experimental stroke has been shown to be injurious to the heart. However, whether unconditioned neuronal progenitor cell derived-exosomes can instead protect myocardium represents a profound research gap. We investigated the effects of unconditioned neural stem cell derived exosomes as post-injury treatment for cardiomyoblasts from three neuronal culture conditions; adherent cultures, neurosphere cultures and bioreactor cultures. Small extracellular vesicles were enriched with serial ultracentrifugation, validated via nanoparticle tracking analysis, transmission electron microscopy and Western blot analysis prior to utilization as post-injury treatment for H9c2 cardiomyoblasts following oxygen and glucose deprivation. LDH assay was used to assess viability and Seahorse XF high-resolution respirometry analyzer to investigate post-injury cardiomyocyte bioenergetics. We found no evidence that unconditioned neural stem cell derived exosomes are cardiotoxic nor cardioprotective to H9c2 cardiomyoblasts following ischemia-reperfusion injury. Based on our findings, utilizing unconditioned neural stem cell derived exosomes as post-injury treatment for other organs should not have adverse effects to the damaged cardiac cells.

**Supplementary Information:**

The online version contains supplementary material available at 10.1186/s12868-025-00931-1.

## Introduction

Acute myocardial infarction (AMI) is a common presentation for cardiovascular disease and a leading global cause of death. Surviving patients suffer from reduced function, reduced quality of life, and shortened lifespan that also affect patients’ families [1]. Surgical management of acute myocardial infarction by pharmacological fibrinolysis, percutaneous coronary intervention, or coronary artery bypass grafting can be complemented with pharmacological renin-angiotensin-aldosterone inhibitors, antiplatelet therapy, statin therapy and beta blockers [2, 3]. These interventions result in anti-inflammatory [4], antithrombotic [5], and antioxidant effects [6], reduce endothelial dysfunction [5] and lower cardiac workload [7]. However, approximately one third of patients respond poorly to revascularization surgery despite improved perfusion [8] and/or current pharmacologic therapies. The mechanisms contributing to failed clinical therapy and poor patient outcomes have yet to be fully realized but may be due to mechanisms extrinsic to the myocardium. Furthermore, heart pathologies leading to cardiac arrest is an extreme insult to central nervous system as interrupted cerebral perfusion leads to global cerebral ischemia [9].

The physiologic relationship between the injured brain and injured heart is emerging as a novel therapeutic target for clinical intervention for both AMI and cerebral ischemia. Acute cardiac abnormalities following AMI are associated with an excess risk of subsequent stroke and one of the most feared disabilities in survivors of myocardial infarction is permanent cognitive impairment. The incidence of stroke after acute myocardial infarction ranges from 1 to 2% during the hospital stay with highest incidence in the first days [10]. An opposing link between a stroke and a subsequent cardiac failure is even more evident. Data from stroke patients has revealed that nearly 90% of patients with ischemic stroke have cardiac symptoms up to 4 months following the stroke [11], and 2–6% of heart related mortality occurs 3 months following a stroke [12, 13]. Stroke patients express large amounts of plasma cardiac enzymes such as troponin and creatine phosphokinase which are markers of cardiac cell stress/death, and catecholamines, which are associated with elevated blood pressure under stress conditions [14]. Ishikawa et al.. using in vitro and in vivo approach showed that oxygen-glucose deprivation (OGD)-conditioned primary neuronal cell derived supernatant, and experimental stroke, both independently mediated cell death in the heart [15], suggesting a close pathological link between ischemic stroke-associated secreted signals and AMI. However, whether circulating factors derived from the brain could instead modulate a myoprotective response to ischemic injury has not been investigated and remains a critical knowledge gap and potential novel therapeutic avenue for AMI.

Recent advances in stem cell-derived exosomes have shown promising results. Since some [16] but not all [17] stem cell-based cardiac therapies showed disproportional advantages compared to direct trans-differentiation and grafting, the protective effect is proposed to be mediated by in a secreted fashion [16]. Small extra cellular vesicles, usually termed exosomes, are secreted nanoscale (30–130 nm) extracellular membrane vesicles of endocytic origin that differ from other secreted extracellular vesicles by size, density, lipid, protein and nucleic acid composition [18]. Humoral exosomes are present in the circulation and facilitate intercellular communication, regulating recipient cell function [19]. The therapeutic potential of stem cell-derived exosomes has been reported [17, 20] to reduce infarct size, alter cardiac remodeling, and promote revascularization [21]. Stem cell-derived exosomes can cross the blood-brain barrier, and are transported into cells *via* direct fusion with plasma membrane, or binding to cell surface proteins and adhesion molecules [22]. In the adult brain, vestigial stem cell progenitor cells provide protection and promote neuronal repair after cerebral ischemia *via* exosomes [23].

After ischemia-reperfusion injury the damaged myocardium undergoes degenerative remodeling, leading to irreversible loss of functional cardiomyocyte activity, causing heart failure and death [24]. Stem cell- and cardiac progenitor cell-derived exosomes have been shown to promote cardiomyocyte survival, including exosomes derived from cardiac telocytes, cardiac progenitor cells, embryonic stem cells (ESC), induced pluripotent stem cells (iPSC), mesenchymal stromal cells (MSC), and bone marrow-derived mesenchymal stromal cells (BM-MSC) [25, 26]. Against brain ischemia, unconditioned neural stem cell derived exosomes showed a marked increase in neural cell viability compared to induced pluripotent stem cell derived cardiomyocyte exosomes, and a notable decrease in stroke volume in the brain in a mouse middle cerebral artery occlusion model [27]. On the other hand, ischemia conditioned neuronal cell derived supernatant and experimental stroke was shown to be detrimental to the heart [15]. Thus, the safety of exosomes derived from unconditioned neural progenitor cells for treating postischemic cardiomyocytes, or whether exosomes derived from unconditioned neural progenitor cells can confer cardiac protection, represents a knowledge gap in the development of novel therapeutic drug targets for both stroke and AMI. Since unconditioned neural progenitor cell derived exosomes can be used to treat brain ischemia, but are potentially even detrimental to the heart, we sought to investigate the safety and efficacy of unconditioned neural progenitor cell derived exosomes in treating post-injury cardiomyocytes.

## Materials and methods

**Fig. 1:**
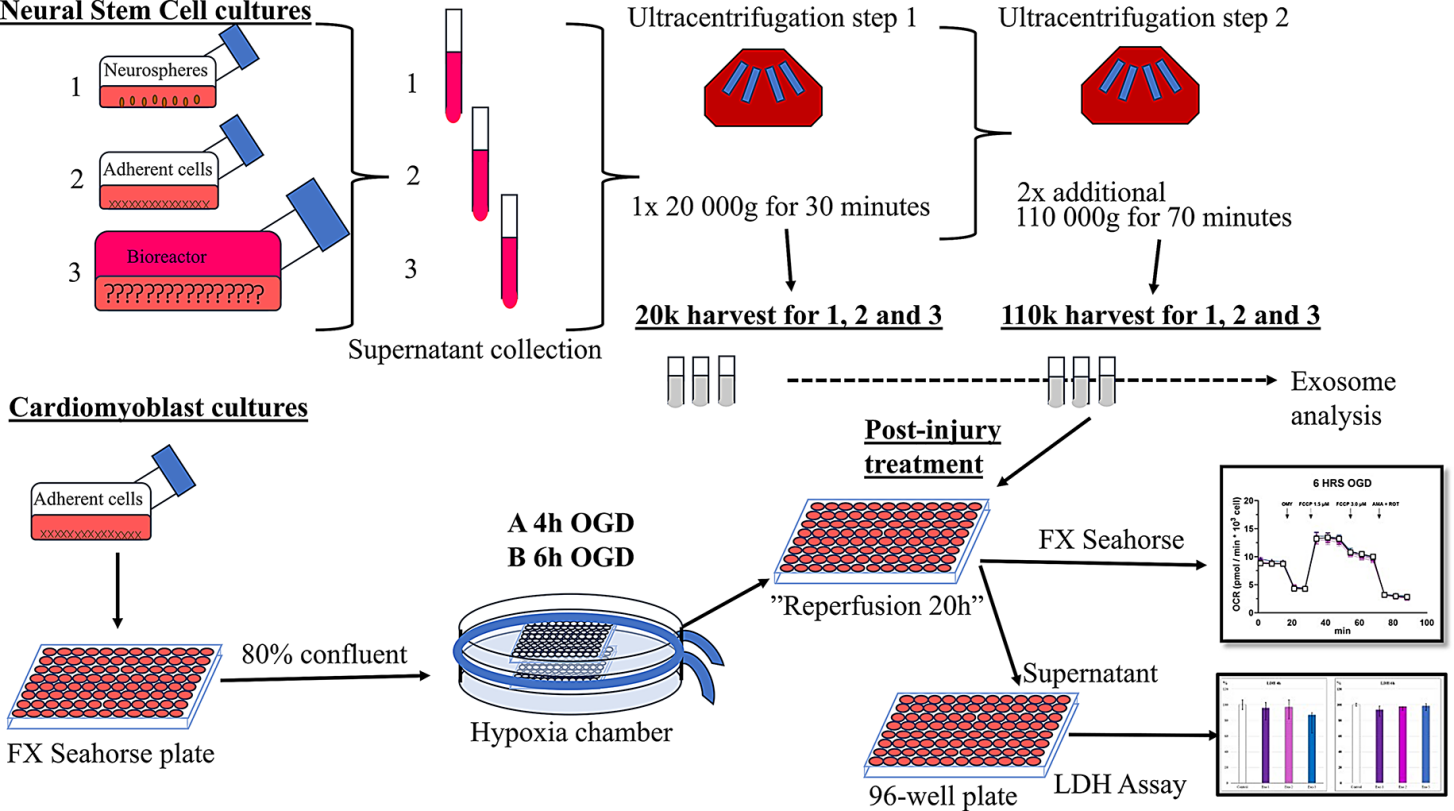
Complete Workflow. Supernatant was collected at multiple time points from 1 neurospheres, 2 adherent neural stem cell cultures, and 3 from cells grown in CL 1000 bioreactor, for serial ultracentrifugation steps. 20k and 110k harvests were resuspended and frozen for further analysis. H9c2 cardiomyoblasts were cultured in FX seahorse plates, subjected to 4-hour and 6-hour oxygen glucose deprivation, followed by treatment with exosomes. Supernatant was collected for LDH assay, and mitochondrial respiration was measured using FX seahorse

The full workflow is depicted in Fig. 1.

### Unconditioned rat fetal neural progenitor cell cultures

Gibco Rat Fetal Neural Stem Cells (NSC) (Cat. No. N7744-100, ThermoFisher) were cultured as adherent cultures, neurosphere suspension cultures and in bioreactors following manufacturer´s instructions. Complete StemPro™ NSC SFM™ (neural stem cell serum free media) medium was used for optimal growth and expansion of Rat Fetal NSCs, and to keep them undifferentiated in all stem cell culture conditions. StemPro™ NSC SFM™ (Cat. No. A1050901, ThermoFisher) complete medium consists of StemPro™ NSC SFM™ supplemented with EGF (Cat. No PHG0314, ThermoFisher), bFGF (Cat. No. PHG0024 ThermoFisher), and GlutaMAX™-I (Cat. No. A1286001, ThermoFisher).

A fresh patch of P0 NSC cell line was removed from liquid nitrogen and thawed under sterile conditions following manufacturer’s instructions and subsequently seeded to 6-well plate and cultured at 37 °C, 5% CO_2_ and 90% humidity until 85% confluent before passages. All cultured cells were re-seeded with fresh complete StemPro™ NSC SFM™ at recommended density of 2 × 10^5^ viable cells per cm^2^. At P3 the NSC were seeded to three different culture conditions in addition to Nunc™ Lab-Tek™ II Chamber Slide™ System (Cat. No 154534PK, ThermoFisher) for phenotype quality control. All NSC and cardiomyocyte cultures were passaged using StemPro™ Accutase™ Cell Dissociation Reagent (Cat. No. A1110501, ThermoFisher), all cell culture handling was performed in a sterile cell culture room in a fume hood, which was sterilized with UV-light daily.

### Adherent culture

For adherent neuronal cultures Thermo Scientific™ Nunc™ Non-treated T75 flasks (Cat. No 156800, ThermoFisher) were coated with CELLstart™ Substrate (Cat. No A1014201, ThermoFisher) prior to seeding. Culture media of 15mL was changed every two days after seeding and then supernatant harvested and media changed every two to three days after 7–10 days, following similar timeline with neurosphere cultures. Condition of the cell culture was inspected daily under microscope.

### Neurosphere culture

For neurosphere cultures the same Thermo Scientific™ Nunc™ Non-treated T75 flasks were seeded without any pretreatment or coating. Culture media of 15mL was changed every two days after seeding and then supernatant harvested and media changed every two to three days after 7–10 days, when neurosphere diameter reached 2–3 mm. Condition of the neurosphere culture was inspected daily under microscope.

### CELLine CL 1000 bioreactor

DWK Life Sciences Wheaton™ CELLine™ Bioreactor Flask (Cat. No 12801803, Fisher Scientific) was inoculated to cell compartment according to manufacturer’s instructions with total volume of 15mL of fresh complete StemPro™ NSC SFM™ with viable NSC at P3. After seeding, 950 mL of Complete StemPro™ NSC SFM™ media was added into the medium compartment and the bioreactor was carefully placed inside incubator. Cell compartment harvest begun at day 7 and every 3–4 days after first harvest for 4 consecutive harvests. Due to the size of the CL 1000 bioreactors and the thick colored medium compartment, we were completely blinded by the set up and could not reliably assess the cells, whether they were growing as neurospheres as intended or as an adherent culture.

### Exosome extraction and characterization

We aimed to utilize serial ultracentifugation to enrich population of small extracellular vesicles 30–120 nm of size from cultured supernatant, further here referred to as exosomes. Following each harvest cycle from each culture condition the exosome extraction and characterization was performed identically to all supernatants [28]. First, 15mL of supernatant from each culture condition was centrifuged at 2500 g for 30 min at room temperature (SL 16R, Thermo Fisher Scientific), and supernatant collected. The aspirated supernatant was then centrifuged in Optima™ L-80 XP Ultracentrifuge (Beckman Coulter) using a Ti 70.1 fixed-angle rotor (Beckman Coulter) at 20 000 g for 30 min at 4 °C. The 20k pellet was resuspended to sterile H_2_O and frozen, and the supernatant collected for two more cycles of 110 000 g for 70 min each, and final pellet resuspended to sterile H_2_O. The 20k and 110k samples were frozen at -80 °C in 100 µL aliquots, and used for experiments after one freeze-thaw cycle.

### Nanoparticle tracking analysis

EV samples (20k and 110k) were analyzed by nanoparticle tracking analysis (NTA) using Nanosight model LM14 (Nanosight) equipped with blue (404 nm, 70 mW) laser and SCMOS camera at EV Core Facility, Department of Biosciences and the Faculty of Pharmacy and Institute for Molecular Medicine Finland, FIMM. The samples were diluted in 0.1 μm filtered (Millex VV, Millipore) DPBS to obtain 40 − 100 particles/view, and record five 30 s videos using camera level 14 with automatic temperature setting of 25 °C. The data were analyzed using NTA software NTA 3.4 Build 3.4.4, with the detection threshold 5 and screen gain at 10 to track as many particles as possible with minimal background.

### Transmission electron microscopy

Electron microscopy was performed at EV Core Facility, Department of Biosciences and the Faculty of Pharmacy and Institute for Molecular Medicine Finland FIMM. Electron microscopy (EM) samples were prepared by negative staining as described earlier [29]. Due to the low NTA value, samples were concentrated using AMICON Ultra 0,5mL concentrators from Merck. The cutoff of the concentrators is 10KDa, and the concentration process was performed following manufacturers recommendations. The total centrifugation time was 45 min, and samples were concentrated 3,4 times (volume was reduced from 100 µl to 30–33 µl). Extracellular vesicles were prepared for EM by loading to carbon coated and glow discharged 200 mesh copper grids with pioloform support membrane. The samples were loaded to the grids without dilution. EVs were fixed with 2.0% PFA in NaPO4 buffer, stained with 2% neutral uranyl acetate, further stained, and embedded in uranyl acetate and methyl cellulose mixture (1.8/0.4%). EVs were viewed with transmission EM using Jeol JEM-1400 (Jeol Ltd., Tokyo, Japan) operating at 80 kV. Images were taken with Gatan Orius SC 1000B CCD-camera (Gatan Inc., USA) with 4008 × 2672 px image size and no binning.

### Western blot

To confirm small extracellular vesicle characteristics a Western blot analysis was performed. Exosomes were loaded into SDS-PAGE gels and transferred to PVDF membrane after gel electrophoresis. 110 kDa ITGB1 Polyclonal Antibody (Cat No. PA5-78028, Thermo Fisher), 25 kDa CD9 Polyclonal Antibody (Cat No. PA5-85955, Thermo Fisher), and 48 kDa TSG101 Polyclonal Antibody (Cat. No. PA5-95851, Thermo Fisher) were used as positive exosome controls and 90kda Calnexin Polyclonal Antibody (Cat. No PA5-34804, Thermo Fisher) as negative control. SuperSignal™ West Femto Maximum Sensitivity Substrate (Cat. No.34095, Thermo Fisher) and ChemiDoc Imaging System (Bio-Rad) were used for detection. The gels were cut in half at 75 kDA prior to incubation with primary antibodies to avoid unspecific binding.

### Cardiomyocyte cultures

A fresh patch of H9c2 (2 − 1) BDIX heart myoblast cells (Cat. No. 88092904, Sigma Aldrich) were thawed according to manufacturer’s instructions. H9c2 cells were seeded in Nunc™ EasYFlask™ Cell Culture Flasks (Cat. No 156499, Thermo Fisher) at P2, and cultured in Dulbecco’s Modified Eagle’s Medium (DMEM) modified to contain 4 mM L-glutamine, 4500 mg/L glucose, 1 mM sodium pyruvate, and 1500 mg/L sodium bicarbonate (Cat. No. 41966052, ThermoFisher), supplemented with, 2 mM Glutamine (Cat. No. A1286001 Thermo Fisher), 10% fetal bovine serum (FBS) (Cat. No. F9665-500ML, Sigma Aldrich), and 1% penicillin and streptomycin (Cat. No. 15140122, ThermoFisher) (complete medium), under 95% air/5% CO2, until 80% confluence. Confluence over 80% could affect the cells bioenergetics and was thus avoided. Cardiomyoblast culture were passaged every 3–4 days. Media was changed every 2–3 days, and the condition of the cells were viewed under microscope daily. P4-P13 were used for all oxygen-glucose deprivation (OGD) experiments.

### Oxygen glucose deprivation

H9c2 cell were seeded to Agilent Seahorse XF Cell Culture 96-well Microplate (Cat. No. 101085-004, Agilent Technologies). On the subsequent days after seeding 4000 H9c2 cells/well, after reaching 70–80% confluency, oxygen-glucose deprivation (OGD) was initiated as follows: Media was carefully aspirated from plates using a serial pipette and each well was washed twice with sterile phosphate buffered saline at 37 °C. To initiate OGD, media was changed to DMEM with no glucose, no pyruvate, and no glutamine (Cat. No. A14430-01, Thermo Fisher) without supplementing FBS, and the plates were subsequently placed in a STEMCELL Technologies Hypoxia Incubator Chamber (Cat. No. 27310, STEMCELL Technologies). Hypoxia incubator chamber was flushed with 95% nitrogen and 5% CO_2_ at 20 l/min for 4 min and sealed to initiate hypoxia according to manufacturer’s guidelines and placed to 37 °C incubator for normothermic oxygen glucose deprivation for 4–6 h. The experiment was repeated 3 times with four-hour OGD, and 3 times with 6-hour OGD, each experiment had 7–10 successful replicates in each experiment. After OGD, the cells were reperfused with complete medium with treatment, and placed back to incubation chamber under 95% air/5% CO_2_ for 20 h, before final vitality analysis and mitochondrial respiration analysis.

### Exosome treatment

The validated three exosome treatments were assigned to study groups as follows: Control group samples were treated with sterile PBS. Exo1 received exosomes from 110k NSC neurosphere resuspended pellet, Exo2 received exosomes from 110k NSC bioreactor resuspended pellet, and Exo 3 received exosomes from 110k NSC neurosphere resuspended pellet, similarly to group 1, but from different culture patch. Particle densities in wells were calculated from NTA results indicating the exosome particle density in our frozen exosome samples diluted to the treatment volumes of 20 µl/well standardizing similar pipetting effects to each well and treatment. The total volume of media + treatment at reperfusion phase was 100 µL/well. First, we injected exosome treatments at 5 µl/well and 15 µl/well (total volume with PBS to 20 µl/well) in addition to 80 µl/well of complete media, to reach particle density of ~ 700 *10^6^ and 2*10^9^ exosomes/mL, respectively for 2 experiments. We then focused on the higher concentration of 15 µl/well (2*10^9^ exosomes/mL) for the rest of the experiment based on literature regarding particle density and effect [30], and due to practicality.

### Seahorse XF– mitochondrial respiration

Mitochondrial respiration was measured with high-resolution respirometry (Seahorse XF 96, Agilent) based cells oxygen consumption rate (OCR), according to manufacturer’s instructions. After the 20-hour reperfusion phase, before the analysis, cell culture media was aspirated for LDH assay to parallel 96-well plate, and replaced with XF Assay Media (Phenol Red-Free Seahorse XF Base Media, 10 mM glucose, 1.25 mM pyruvate and 2 mM glutamine, pH 7.2) and the cells were incubated for one hour in a CO_2_-free incubator (+ 37 °C) to diffuse excess CO_2_ from cells, reagents, and plastics. Directly before the analysis, a fresh de-gassed XF Assay Media was changed to the cells.

The Seahorse Mito Stress Test-analysis was initiated with basal mitochondrial respiration measurement, followed by ATP synthase inhibition with 1 µM oligomycin (OMY) injection. Cells’ maximal mitochondrial respiration capacity was measured after ATP synthase uncoupling with carbonyl cyanide 4-(trifluoromethoxy)phenylhydrazone (FCCP) titration. Finally, 0.5 µM antimycin A and 0.5 µM rotenone (AMA + ROT) injection was used to block mitochondrial complexes III and I, resulting to electron transport chain shutdown. The residual OCR after AMA + ROT injection is assumed to occur due to non-mitochondrial OCR and subtracted from total OCR values to calculate basal and maximal respiration. OCR after 1.5 µM FCCP injection was used to maximal respiration capacity calculation. Data was normalized to number of cells, quantified based on nuclei staining by cell membrane permeable staining dye (Hoechst 33342, 0.5µM) and cells were imaged with an automated imaging system, ImageXpress Pico (Molecular Devices) (Data supplement 1).

Statistical analysis One-Way ANOVA multivariate with Dunnett’s test. P-value 0.05 or lower (*p* ≤ 0.05) was set as a threshold for statistically significant difference level. Confidence level was set to 0.05 (ɑ ≤ 0.05).

### Cell viability assay

Post-injury extracellular release of lactate dehydrogenase (LDH) was measured to assess the relative viability between study groups as previously done [27]. In brief, Invitrogen™ CyQUANT™ LDH Cytotoxicity Assay (Cat. No. C20301, Thermo Fisher) was used according to manufacturer’s instructions to measure 490 nm and 680 nm absorbances (LDH activity) with a plate reader (EnSpire Multimode Plate Reader, Perkin Elmer) in the medium at 20 h of reperfusion and compared between groups. Absorbance measured at 680-nm (background) was subtracted from the 490-nm absorbance, divided by number of cells in the wells. Control group measurement was set as 100%, and study group measurements are presented as percentage of control group values. Statistical analysis One-Way ANOVA multivariate with Dunnett’s test. P-value 0.05 or lower (*p* ≤ 0.05) was set as a threshold for statistically significant difference level (IBM SPSS Statistics 28). Confidence level was set to 0.05 (ɑ ≤ 0.05).

### Immunofluorescence

Glial fibrillary acidic protein (GFAP) antibody (Cat. No PA5-16291, Thermo Fisher), Doublecortin (DCX) antibody (Cat. No 48-1200, Thermo Fisher), Nestin antibody (Cat. No. PA5-79729, Thermo Fisher), and GalC (Cat. No. PA5-109753, Thermo Fisher) were utilized to demonstrate astrocytic, neuronal, undifferentiated neural stem cells, and oligodendrocytic characteristics, respectively, as we have done before [31]. In brief, the cells cultured in chamber slides were washed in PBS twice for 15 min at room temperature. After a 10 min permeabilization step in 0.1% Triton x-100 in PBS, the sections were washed for 5 min in PBS, then blocked with 5% Bovine serum albumin in PBS at 4 °C overnight. The chamber slides were washed with PBS twice, followed by incubation in above mentioned primary antibody solutions (Nestin 1:400, GFAP 1:200, DCX 1:133, Galc 1:200) in 1% BSA in PBS at 4 °C overnight. The following day the chamber slides were washed three times in PBS for 5 min, followed by secondary antibody solution of Goat anti-Rabbit IgG (H + L), Superclonal™ Recombinant Secondary Antibody (Cat. No. A27034, Thermo Fisher) [1:1000] in PBS, at 4 °C overnight. The chamber slide system was finally washed two more times with PBS, then disassembled prior to mounting with DAPI, and imaged with Zeiss AxioImager 1 (Zeiss), with Zeiss Zen 2 software.

### Power calculations

We performed power calculations based on previous LDH assay data from Sun et al. [27]: The calculated sample sizes for each condition were 9 biological replicates in addition to technical replicates (expected mean incidences 50% ±15% and 70%, alpha 0.05, beta 0.2, power 0.8).

## Results

### NSC characteristics

**Fig. 2:**
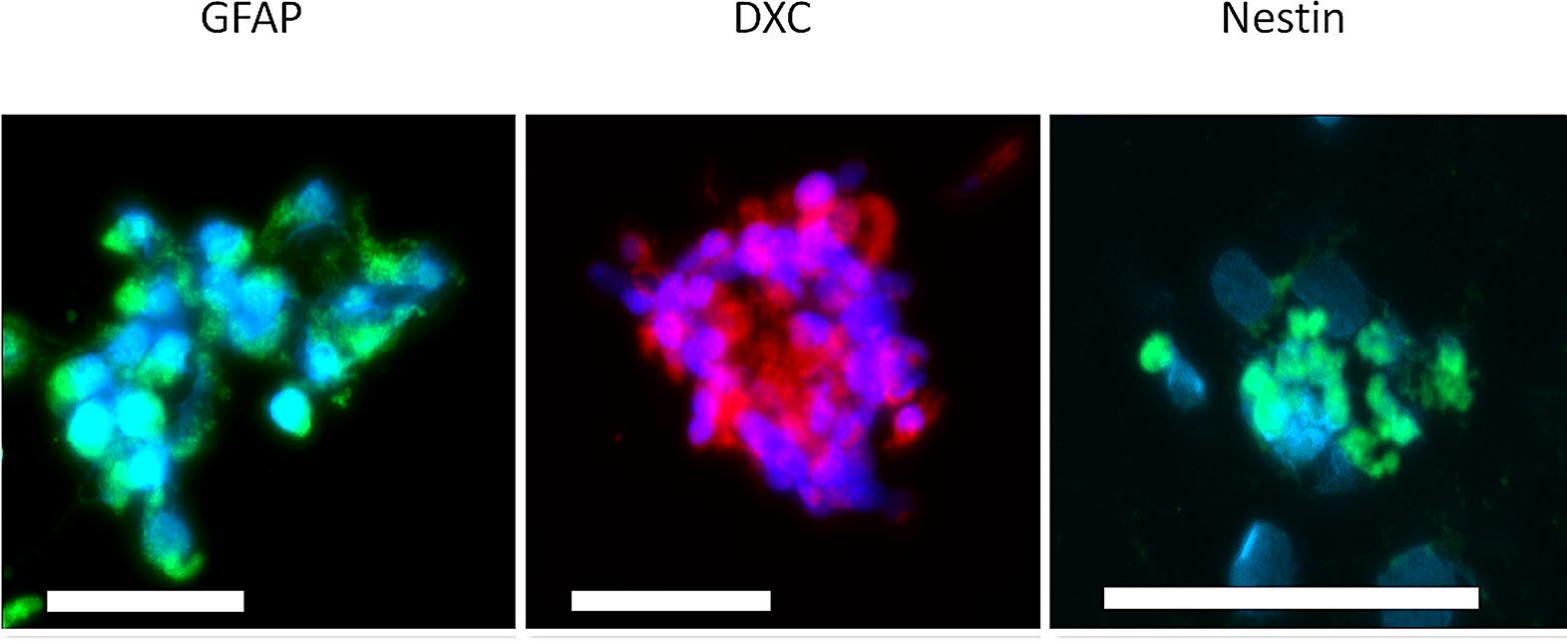
Neural stem cell phenotype quality control. At day 3 in a chamber slide culture, there were groups of cells forming neurosphere like gatherings, expressing GFAP (green), DXC (red) and Nestin (green), and DAPI (blue), indicating the progenitor cells expressed characteristics of neural stem cells, neurons and astrocytes early in the process. Scale bar is 100 μm

**Fig. 3:**
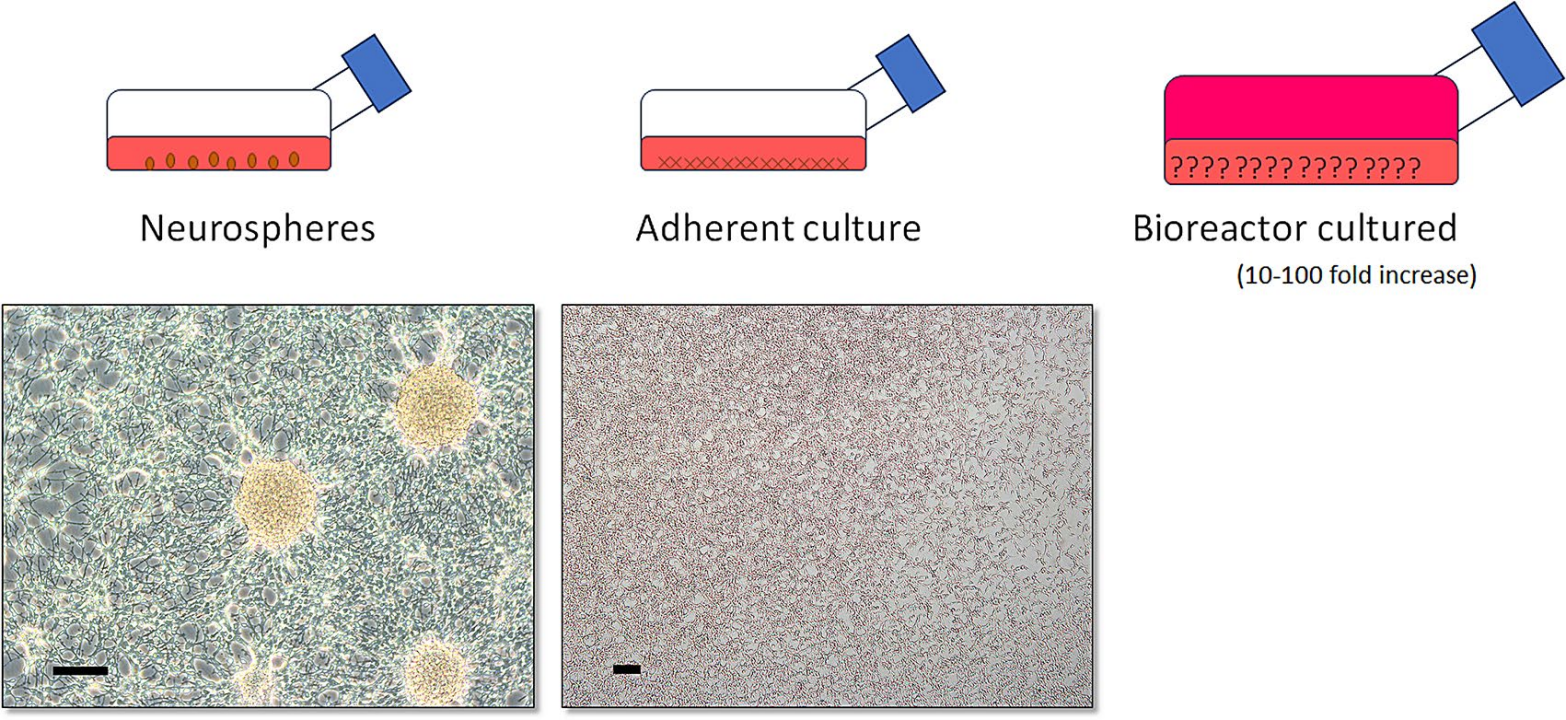
Culture growth conditions. The cells were cultured as neurospheres and adherent cultures, and in one liter CL1000 bioreactor. Neurosphere cultures showed typical floating spheres, which later upon touching the flask bottom attached to the bottom and continued growing. There were no spheres in adherent culture flask. We could not investigate the conditions inside CELLline CL 1000 bioreactor. Scale bar is 200 μm

Immunofluorescence was performed early in the study to determine successful thawing of neural stem cells and characteristics of newly forming neurospheres. Close to 90% of cells in chamber slide cultures expressed Nestin after thawing indicating the cells being undifferentiated, which is in accordance to manufacturer (Data not shown). The cells in small chamber slide chambers showed uneven spread, and clumps of cells started forming neurosphere-like gatherings 3 days after seeding. These clumps of cell expressed GFAP, DCX and Nestin (Fig. 2). Galc stainings were completely negative in all of samples (Data not shown). In the T75 flasks, the cell cultures showed expected macroscopic neurosphere and adherent culture characteristics. We could not validate the culture growth inside the bioreactors (Fig. 3).

### Exosome validation

**Fig. 4:**
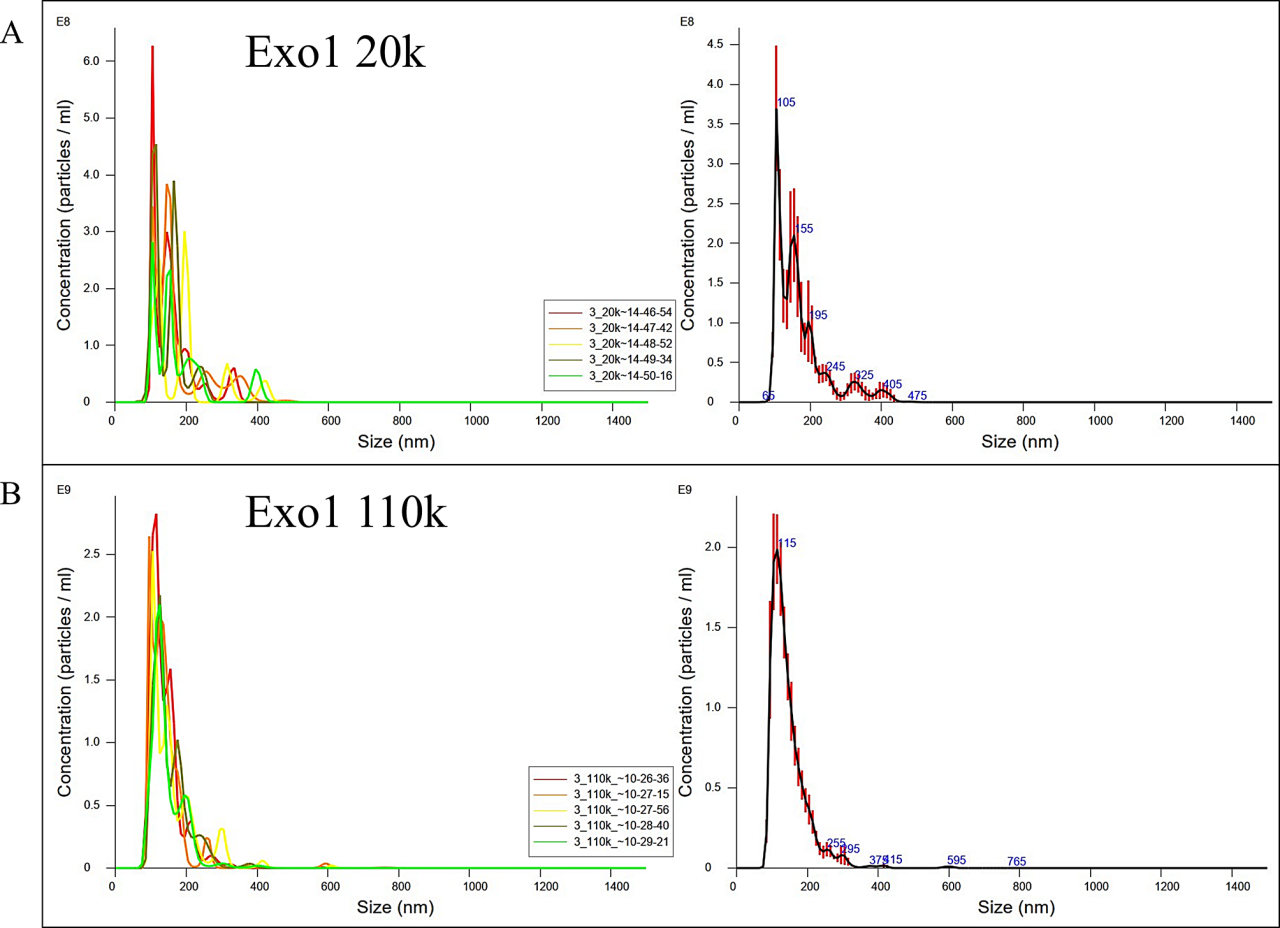
Nanoparticle tracking analysis. A representative image from Nanoparticle Tracking Analysis from the same sample showing (**A**) 20k yield and (**B**) 110k yield, from neurosphere culture labeled Exo1. The particle density was higher in 110k samples, with more small EVs. The left side represents technical NTA replicates for each measurement from a single sample. The rights side represents averaged finite track length adjustment (FTLA) Concentration / Size graphs for NTA analysis. Error bars indicate ± 1 standard error of the mean

Exosomal cargo is known to be dependent on not only cell culture conditions [32], but also on the extraction method [28]. We took extra care to validate our exosome yield, before continuing with the experiment. All harvested exosomes in all culture conditions, and both 20k and 110k ultracentrifuged subpopulations were examined for particle density. Nanoparticle tracking analysis returned notable exosome yields in all of our samples representing expected distribution [28, 32], as illustrated in Fig. 4, and in Tables 1 and 2. We managed to successfully utilize serial ultracentifugation to enrich population of small extracellular vesicles in 110k pellet, as seen in Table 1.

**Table 1:** Descriptive characteristics of all exosome harvests from resuspended 20k pellet and 110k pellet, and statistics between the two ultracentrifugation steps. The NTA analysis revealed expected small extracellular vesicle characteristics in the 110k pellet, confirming properly enriched EV population compared to the 20k pellet

Variable	Group	N	Mean	Std. Deviation	p (Independent samples t-test)
Concentration (particle/mL)	20k	8	0,40*10^10^	28,67	0,038
	110k	8	0,92*10^10^	56,62	
mean (size, nm)	20k	8	169,13	14,84	0,032
	110k	8	153,88	10,32	
mode (size, nm)	20k	8	134,26	15,83	0,013
	110k	8	116,95	6,49	
D10 (size, nm)	20k	8	106,81	6,56	0,348
	110k	8	103,90	5,38	
D50 (size, nm)	20k	8	153,65	15,92	0,02
	110k	8	137,13	7,79	
D90 (size, nm)	20k	8	264,64	37,71	0,044
	110k	8	229,41	24,43	
SD (size variability, nm)	20k	8	70,00	10,97	0,049
	110k	8	58,31	10,71	

**Fig. 5:**
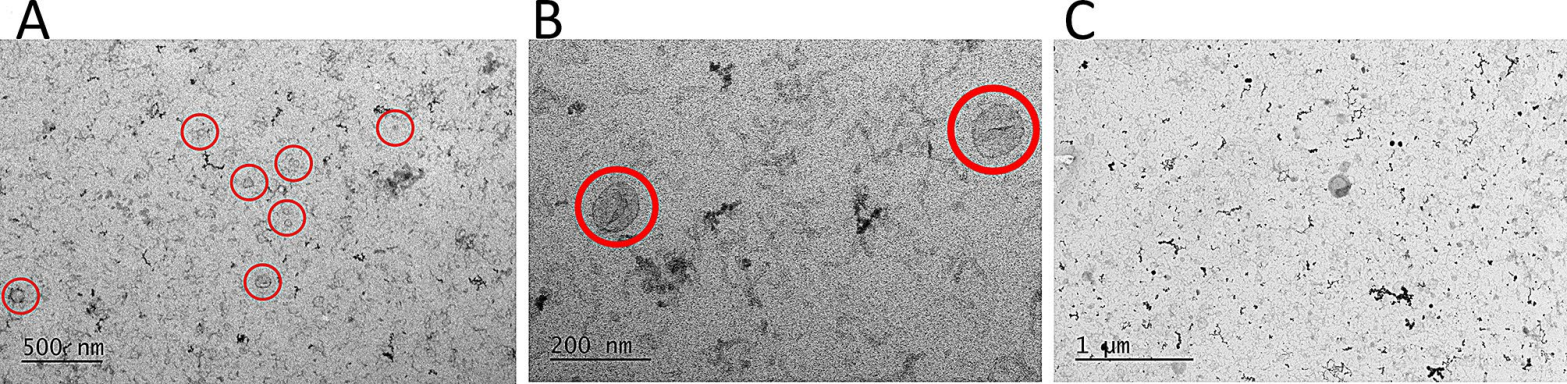
Electron microscopy. A representative image from electron microscopy findings. **A** and **B** show typical yeild of small extracellular vesicles (red circle) from Exo1 sample, neurosphere culture 110k. **C**. EM showed some atypical structures on the background in all samples (dark string like structures)

An aliquot of all exosomes were then sent for transmission electron microscopy for imaging confirmation. Electron microscopy was performed by a blinded analyst. All samples presented EVs of typical morphology and variable size, however the main population consisted of small EVs. Very large EVs (roughly 1 μm or above) were not observed. EM showed some atypical structures on the background in all samples. Figure 5.

**Fig. 6:**
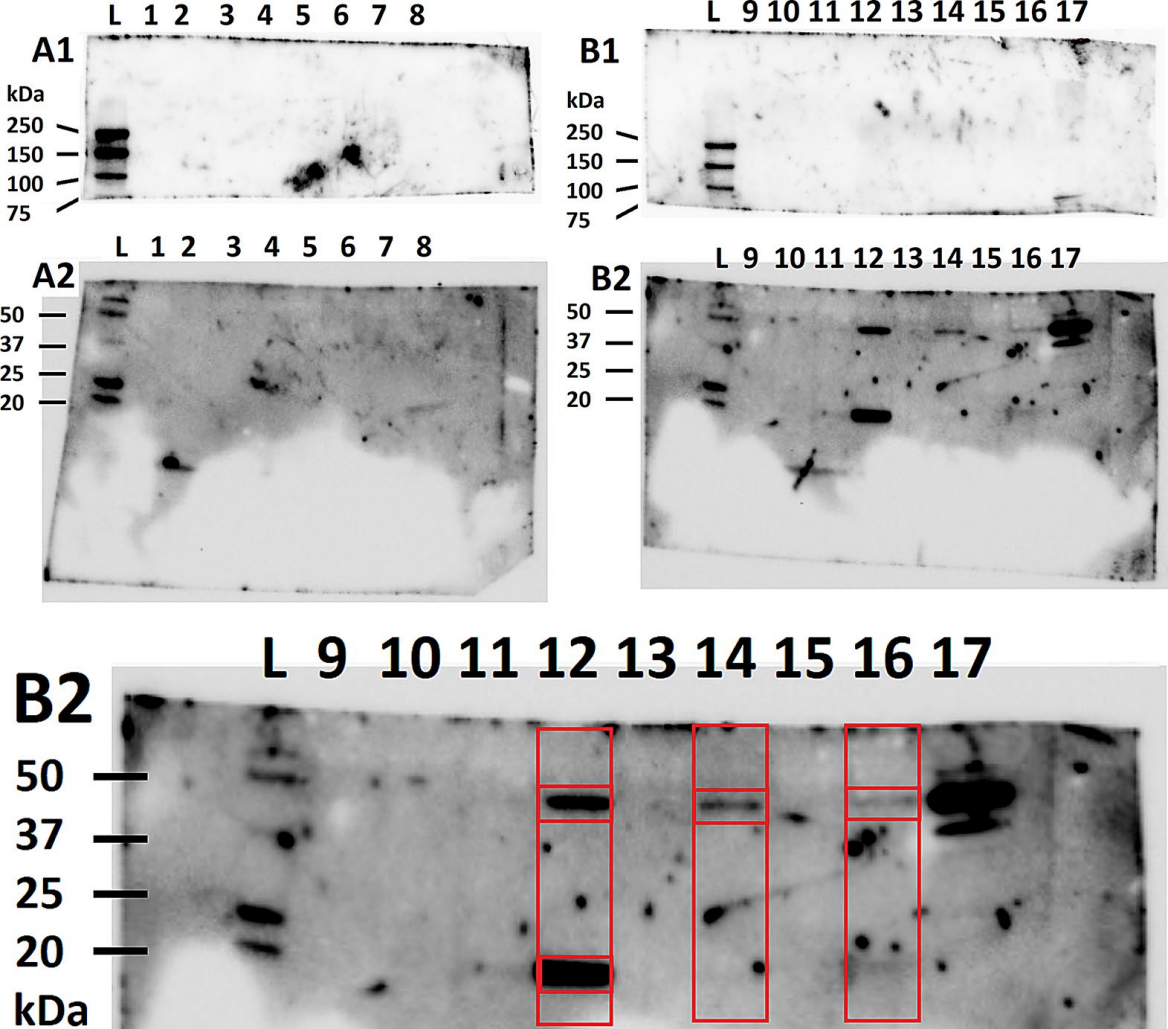
Western blot. A1 and A2 are gels from the samples 1–8, B1 and B2 are gels from samples 9–16, and sample17 is technical control showing positive for calnexin at 90 kDa (negative antibody control, B1, line 17) and TSG101 at 25 kDa (positive control, B2, line 17). L stands for ladder. 1 = Bioreactor 20k, 2 = Bioreactor 110k, 3 = Neurosphere 20k, 4 = Neurosphere 110k, 5 = Adherent cells 20k, 6 = Adherent cells 110k, 7 = Bioreactor 20k, 8 = Bioreactor 110k, 9 = Adherent cells 20k, 10 = Adherent cells 110k, 11 = Neurospheres 20k, **12 = Neurospheres 110k**, 13 = Bioreactor 20k, **14 = Bioreactor 110k**, 15 = Neurosphere 20k, **16 = Neurosphere 110k**, 17 = control. Only three samples of our exosomes returned positive findings in Western blot analysis. These were labeled as our study groups as follows: 12 = Exo 1 Neurospheres 110k, 14 = Exo 2 Bioreactor 110k and 16 = Exo 3 Neurospheres 110k

We then pursued to validate exosomal protein markers with Western blot for suspected positive and negative markers [33]. Out of all 16 samples quantified to have small extracellular vesicles determined by NTA analysis and electron microscopy, only three also returned positive protein markers (Fig. 6).

None of the samples from adherent neuronal stem cell cultures expressed expected exosome markers. The three samples that also had expected exosome proteins were from 110k neurosphere cultures (x2) and 110k bioreactor culture. TSG101 was positive in all the three samples, and CD9 in one of the 110k neurosphere cultures. ITGB1 and Calnexin were negative in all samples, including the three otherwise positive. These exosome markers were chosen according to MISEV guidelines (Thery et al. 2018), which also state that due to limited but rising knowledge we cannot be sure whether all markers are expressed, and negative markers are surely negative in all exosomes. We decided to continue the experiment only with the exosome lineages with confirmed positive findings for NTA analysis, Electron microscopy and Western blot. Due to the differential CD9 expression, we termed the groups Exo1 (110k neurospheres), Exo2 (110k bioreactor), and Exo3 (110k neurospheres), in which the exosome marker CD9 was expressed only in Exo1. The descriptive statistics of NTA findings of Exo1, Exo2, and Exo3 are shown in Table 2. The size distribution varied significantly between the exosome groups. However, they all present the expected size distribution of small extracellular vesicles referred to as exosomes.

**Table 2:** Descriptives and statistics of the treatment groups as for the entire set of NTA derived descriptors. The exosome populations differed in all of the measured parameters. Mean (nm) and mode (nm) are parameters describing the diameter of exosomes and their frequency distribution in the sample. D10, D50 and D90 are dimensional parameter that indicate that 10%, 50% and 90% respectively of the exosomes are included below the corresponding diameter within the sample. Statistical analysis was performed as Student t-test

Variable	Group	Mean	Standard Error	*p* (t-test)
Concentration (particle/mL)	Exo1	1,5*10^10^	8,35*10^8^	0,008
	Exo2	1,36*10^10^	9,22*10^8^	
	Exo3	1,84*10^10^	1,74*10^9^	
mean (size, nm)	Exo1	146,7	3,8	< 0,001
	Exo2	160,5	3,1	
	Exo3	148	5,8	
mode (size, nm)	Exo1	112,3	4,9	0,03
	Exo2	124,5	5,0	
	Exo3	103,6	0,6	
D10 (size, nm)	Exo1	100,3	1,6	0,001
	Exo2	107,4	1,3	
	Exo3	94,3	0,5	
D50 (size, nm)	Exo1	132,5	2,5	< 0,001
	Exo2	141,4	1,5	
	Exo3	127,6	4,2	
D90 (size, nm)	Exo1	208,3	10,0	0,002
	Exo2	233,1	7,5	
	Exo3	242,5	20,8	
SD (size variability, nm)	Exo1	53,2	3,8	0,007
	Exo2	68,2	8,0	
	Exo3	69,1	11,0	

### Cardiomyoblast viability and mitochondrial respiration

**Fig. 7:**
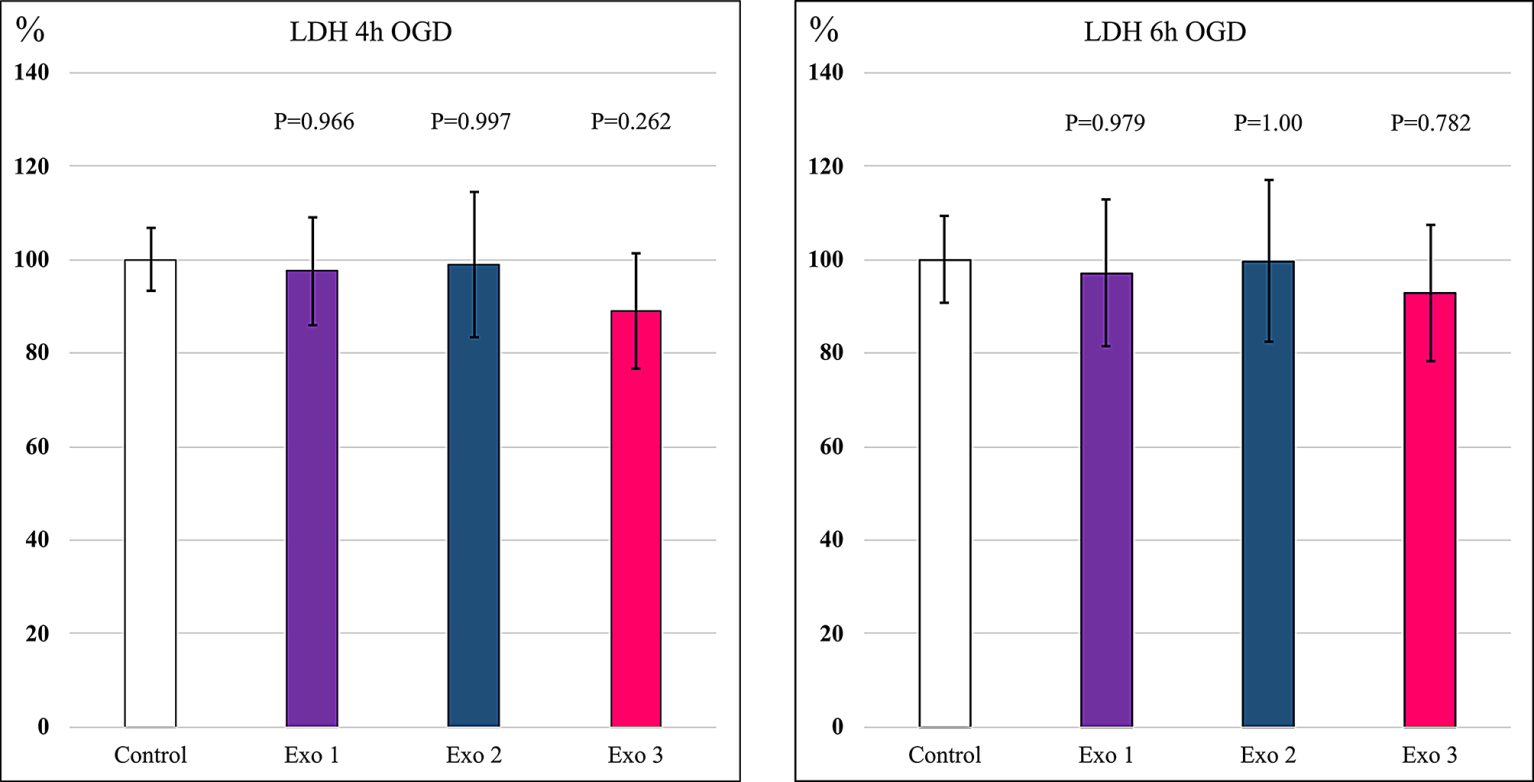
LDH assay. LDH release at 20 h post treatment with exosomes. The results are expressed as means. Error bars show 95% confidence interval. Statistical analysis was performed as One-Way ANOVA multivariate with Dunnett’s test. P-value 0.05 or lower (*p* ≤ 0.05) was set as a threshold for statistically significant difference level

The LDH assay did not reveal any cardiotoxicity nor cardioprotection by the exosome treatment. The LDH released from control group was set as 100%. LDH assay at 20 h after four-hour OGD showed a trend toward cardioprotection with Exo 3 treatment measuring 88.9% compared to control group. This finding did not reach statistical significance, however (*p* = 0.262). We repeated the experiment three times with a six-hour OGD. The exosome treatments showed overall similar LDH release profiles with four-hour and six-hour OGD. There was noticeable variability between wells within the groups. Exo 3 group measured 92.9% LDH release compared to control group after six-hour OGD (*p* = 0.782) (Fig. 7).

**Fig. 8:**
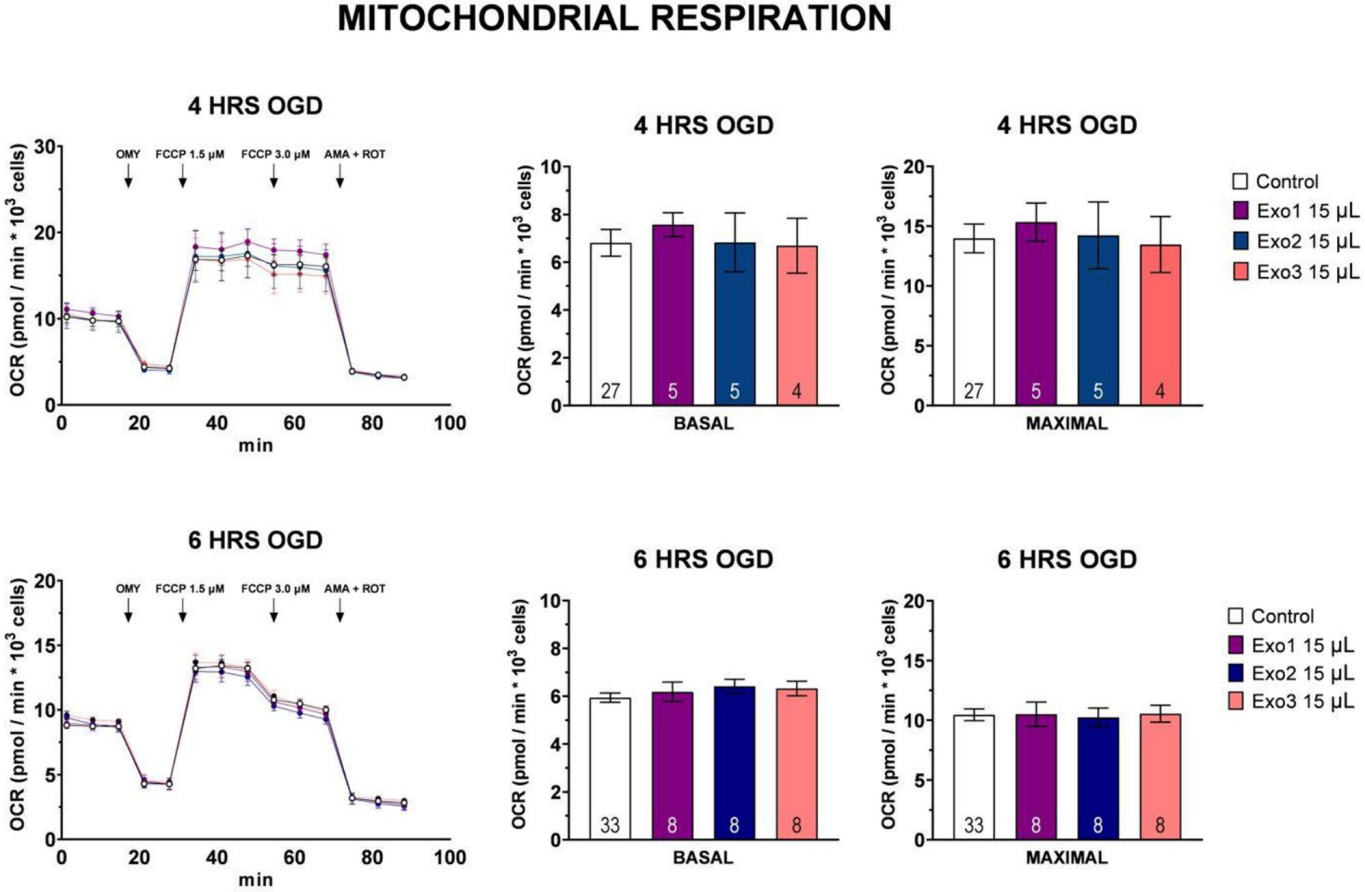
Mitochondrial respiration. Data presented as mean +- Standard Error of Mean (SEM). Statistical analysis with One-Way Anova with Dunnetts test, signifigance level *p* < 0.05, no statistical differences observed in treatment groups when compared to the control group

In line with the viability results, high-resolution respirometry did not reveal any differences in basal or maximal respiration rates between control and exosome-treated groups (Fig. 8). Six-hour OGD resulted in overall lower basal and maximal respiration compared to four-hour OGD. All samples in all experiments followed similar respiratory curve.

## Discussion

In this study, we assessed in vitro the effect of rat NSC-derived exosomes as post-injury treatment in rat H9c2 cardiomyoblast cultures subjected to ischemia-reperfusion injury. With this experimental setup we did not find any evidence of cardiotoxicity nor cardioprotection by the NSC-derived exosomes. These observations bring more insight to treating ischemic brain and heart in a clinical setting, where cardiac arrest results in simultaneous cardiac injury and ischemic brain injury. Current treatment strategies for patient following successful resuscitation and/or coronary artery revascularisation often require intensive care, where post resuscitation care aims to control depth of sedation, body temperature, and mean arterial pressure, and blood gases as secondary prevention [34, 35]. To date, there are no drug therapies targeting both the heart and the brain after ischemia-reperfusion injury. This in vitro experimental set up mimicked clinical ischemia-reperfusion scenario where exosomes could be administered as an adjuvant therapy following coronary artery revascularisation or administration of thrombolytics, or after return of spontaneous circulation in a cardiac arrest patient, targeting the brain and heart after global ischemia. We found no in vitro evidence that treating the brain in a clinical setting with NSC derived exosomes could be harmful for cardiomyocytes after IR-injury. However, the exosome treatment did not improve mitochondrial respiration or cell viability in cardiomyoblasts after ischemia-reperfusion injury. These observations need to be validated further in a pre-clinical rodent cardiac arrest model implementing MISEV-guidelines [33] and stem cell therapies as an emerging paradigm in stroke (STEPS) recommendations [36] before advancing to clinical studies.

Rat H9C2 (2 − 1) Cell Line (cardiomyoblast) was chosen as the cell line as it best mimics cardiomyocyte bioenergetic response to ischemia-reperfusion injury, even without differentiation protocol towards cardiomyocytes [37]. Watkins et al.. demonstrated similarities in H9C2 cell line with human neonatal cardiomyocyte cells, and demonstrated very similar hypertrophic response in H9C2 and PNMC cells to hypertrophic agents in hypertrophy models, and concluded supporting the use of H9C2 in place of primary cardiomyocytes in most hypertrophic assays [38]. Felemban et al. noted disruption of both H9C2 morphological changes and human induced pluripotent stem cell (hiPSC)-derived cardiomyocyte morphological changes after sublethal exposure to phenyl saligenin phosphate, demonstrating similarities between the cell types in response to the triggering agent [39]. Lundy *et* al demonstrated that hiPSC-derived cardiomyocytes express cardiac structural biomarkers which are comparable to adult human cardiomycytes. Based on the literature H9C2 cell line is suitable to model human cardiomyocyte injury model [40]. We took extra care to repeat all experiments with similar confluency since a higher degree of confluence is associated with lower mitochondrial respiratory activity which might alter the results of this study [37]. Based on findings from Kuznetsov et al., ischemia-reperfusion injury for 16 + 6 h would produce 50–60 viability compared to controls. On the other hand, microvesicle uptake is shown to follow almost exponential pattern during time scale of 20–30 h [30], and this is why we chose 20-hour reperfusion/treatment phase, and particle density of ~ 2*10^9^ exosomes/mL. However, during our preliminary set up testing the cultures could not withstand the 16 h ischemia followed by 20-hour reperfusion but started swelling and detaching the 96-well-plate during late reperfusion phase. After preliminary serial time course testing, we ended up with current experimental set up of 4 h and 6 h ischemia, followed by 20-hour reperfusion.

More than 30% of cardiomyocyte volume consists of spatially precisely coordinated mitochondria [41]. Heart’s energy is stored in adenosine 5′-triphosphate (ATP) and in creatine phosphate (PCr) and cardiomyocytes utilize 90% of this energy through oxidative phosphorylation in mitochondria. Exercise is known to cause intermittent decreases in glycolytic activity which is required for physiological cardiac remodeling. Metabolic flexibility is paramount to maintain mitochondrial health in cardiomyocytes [42]. However, prolonged decrease of oxygen, and substrate delivery to cardiomyocytes due to restriction in coronary artery blood flow leads to biochemical energetic failure and pump dysfunction [43]. O_2_ consumption rate is known to be a limiting factor in failing myocardium [44, 45], and more recently exosome cargo has been shown to alter oxygen consumption in an obesity mouse model [46]. Though cardiomyocytes generally utilize free fatty acids as their main bioenergetic substrate, upon injury they shift to glucose-derived metabolism [47]. Hence, in this study we focused on evaluating the impact of OGD-to glucose driven mitochondrial metabolism. Unlike cardiomyocytes which can store glucose as glycogen, neurons rely on constant supply of oxygen and glucose from circulation in addition to intercellular dependence on secreted factors from neighboring resident cells. We hypothesized that NSC derived exosomes would improve cardiomyocyte O_2_ consumption. However, our data does not support this hypothesis, and the possible myoprotective effect seems to be mediated in another manner. In our experimental model, we did not observe improved viability after I/R-injury by NSC exosome treatment either. Viability was assessed indirectly utilizing LDH assay from supernatant collected from the same plates, which were analysed simultaneously at FX Seahorse. There were no differences in mitochondrial basal or maximal oxygen consumption rates, and thus no differences in reserve respiratory capacity between the groups in our study, which has been shown to correlate with cell survival after hypoxia [48].

Previously Ishikawa et al. showed in vivo and in vitro, how experimental stroke were detrimental to the healthy myocardium [15]. Unconditioned NSC -derived exosomes have been shown to be neuroprotective [27], so this finding produced a clinical challenge considering the safety of treating ischemic brain without harming the myocardium. In the new light of this study, we found no evidence of cardiotoxicity with unconditioned NSC derived exosomes. Since there are limited data considering small EV cargoes in post-injury treatments, we were meticulous to validate our exosomes prior to continuing with the experiment, and to avoid false positive or false negative findings mediated by other circulating factors. We followed the MISEV-guidelines to validate our exosome yield, and only continued our experiment with exosomes that matched the full criteria. The exosomes were harvested from NSC cultures with expected immunofluorescence. Galc stainings were negative suggesting there were no oligodendrocyte characteristics early in our cell culture.

This study has multiple limitations. Since ischemic neuronal exosomes can mediate cardiotoxicity, we were careful with media change, and harvest intervals avoiding any possibility for suboptimal nutrition, that could impact the exosome cargoes. This likely resulted in lower than anticipated exosome yields, and possibly the exosome cargoes per se [32]. Unbeknownst to the concentration of our exosome yield we froze our exosomes as 100 µl aliquots before analyzing our exosome characteristics. This resulted in sub-optimal limitation regarding exosome particle density. Frozen exosomes withstand one freeze-thaw cycle relatively well, but multiple freeze-thaw cycles affect the small EVs resulting in unreliable results [49]. Thus, we used one 100 µl aliquot of exosomes in each experiment leading to small replicate number of a preliminary 5 µl treatment group (~ 700 *10^6^ exosomes/mL). We continued the experiment with injecting 15 µl/well (~ 2*10^9^ exosomes/mL) for more reliable data through rest of the experiments [30], and the 5 µl treatment group was discontinued due to statistically insignificant n size. For this reason, we could not present a dose-response curve. Majority of our harvested exosomes samples showed positive NTA and EM results but did not express expected exosomic proteins in Western blot analysis. After thorough consideration we decided to be cautious and only study the effects of exosomes with expected protein signatures, discarding majority of our samples from further experiments, which further limited our experiment. Additionally, we did not comprehensively analyze the studied exosomal cargoes, including proteins, RNAs, and other bioactive molecules. Had there been any differences between the study groups in cardiac protection or cardiac toxicity, it would have been the next logical step to study the differentiation patterns in each culture condition more thoroughly and validate the exosome proteomics in a subsequent phase. Due to aforementioned limitations in exosome yields, we have only studied cell viability indirectly via LDH assay and mitochondrial bioenergetics measurements. Based on our power calculations this study protocol should have detected differences in LDH-release and based on literature differences in viability would have been evident in the oxygen consumption rate measurements as well [48]. Furthermore, electron microscopy showed atypical structures in all samples. We have no explanation for these findings; however we postulate they did not have an effect on the study. Finally, we focused on OGD-to glucose driven mitochondrial metabolism, and possible differences in fatty acid oxidation could not be noticed in this experimental set up.

## Electronic supplementary material

Below is the link to the electronic supplementary material.

Supplementary Material 1: Seahorse XF high-resolution respirometry data was normalized to number of cells, quantified based on nuclei staining by cell membrane permeable staining dye (Hoechst 33342, 0.5μM) and cells were imaged with an automated imaging system, ImageXpress Pico (Molecular Devices) (Data supplement 1)

## Data Availability

No datasets were generated or analysed during the current study.
