# Supplementary figures and images for: Neural progenitor cell-derived exosomes in ischemia/reperfusion injury in cardiomyoblasts
Source: BMC Neurosci. 2025 Feb 5;26:11. doi: 10.1186/s12868-025-00931-1 (PMC11800440; doi:10.1186/s12868-025-00931-1)

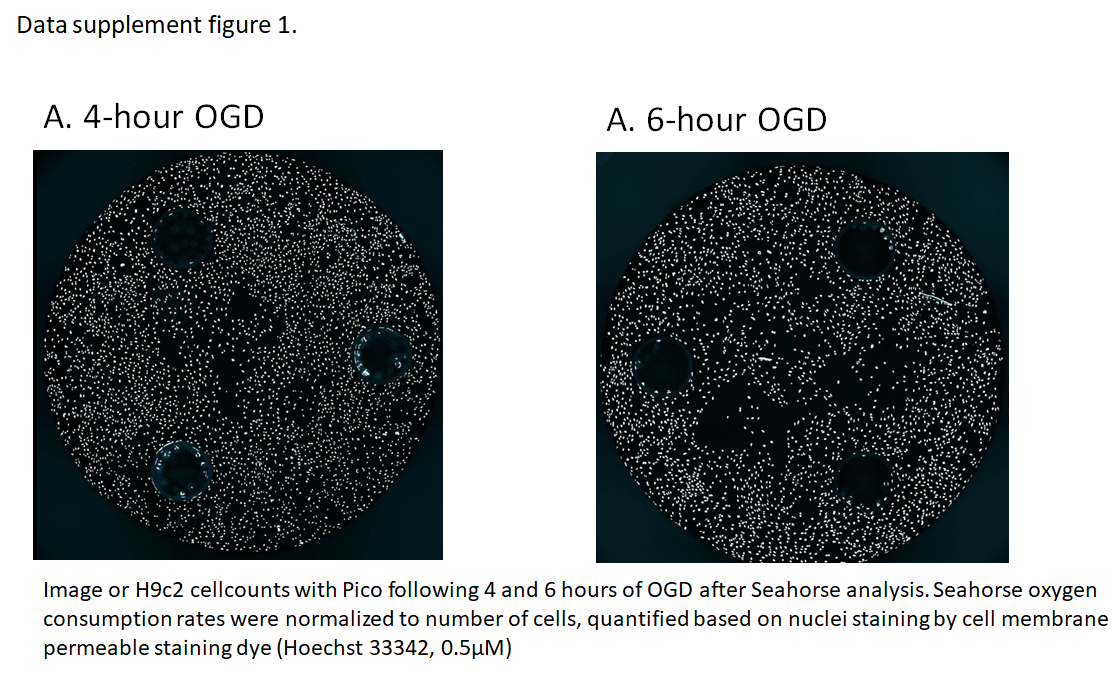

Supplement: Supplementary file 1 — Supplementary Material 1: Seahorse XF high-resolution respirometry data was normalized to number of cells, quantified based on nuclei staining by cell membrane permeable staining dye (Hoechst 33342, 0.5μM) and cells were imaged with an automated imaging system, ImageXpress Pico (Molecular Devices) (Data supplement 1) [file 12868_2025_931_MOESM1_ESM.png]
